# Reliability and validity of the Turkish translation of the MOS-HIV health survey in people living with HIV

**DOI:** 10.3389/fpubh.2026.1966184

**Published:** 2026-10-06

**Authors:** Recep Balık, Seniha Şenbayrak, Ercan Yenilmez, Ayça Aydın, Derya Öztürk Engin, Fatma Yılmaz Karadağ, Özge Çaydaşı, Semiha Çelik Ekinci, Nagehan Didem Sarı, Selami Erdinç, Nurgül Ceran

**Affiliations:** 1 Haydarpaşa Numune Eğitim ve Araştırma Hastanesi, Infectious Diseases and Clinical Microbiology, İstanbul, Türkiye; 2 Fatih Sultan Mehmet Eğitim ve Araştırma Hastanesi, Infectious Diseases and Clinical Microbiology, İstanbul, Türkiye; 3 GATA Haydarpaşa Eğitim Hastanesi Infectious Diseases and Clinical Microbiology, İstanbul, Türkiye; 4 Sancaktepe Şehit Prof. Dr. İlhan Varank Eğitim ve Araştırma Hastanesi, Infectious Diseases and Clinical Microbiology, İstanbul, Türkiye; 5 İstanbul Eğitim ve Araştırma Hastanesi, Infectious Diseases and Clinical Microbiology, İstanbul, Türkiye

**Keywords:** health-related quality of life, HIV, MOS-HIV, reliability, validity

## Abstract

**Background:**

Health-related quality of life (HRQoL) is a key patient-centered outcome in HIV care, particularly in the current treatment era in which virological control is increasingly achievable but residual physical, mental, and social burden may persist. However, the clinical applicability of HIV-specific HRQoL instruments in Turkish-speaking people living with HIV (PLWH) remains insufficiently established. We aimed to characterize HRQoL in a multicenter Turkish sample of PLWH and to evaluate whether the Turkish version of the Medical Outcomes Study HIV Health Survey (MOS-HIV) provides reliable and valid scores for use in this population.

**Methods:**

In this multicenter cross-sectional study, PLWH receiving care at five tertiary training and research hospitals in Istanbul completed the Turkish MOS-HIV. Cognitive debriefing was performed in 20 participants, and 244 were included in the main analysis. Domain and summary scores were calculated according to the user’s manual. Internal consistency was assessed in all 244 participants and test–retest reliability in a subsample of 105 after a median of 15 days. Structural validity was examined using domain-level confirmatory factor analysis and known-groups comparisons were conducted according to comorbidity status, CD4 category, history of AIDS-defining illness, and number of tablets in the treatment regimen.

**Results:**

Participants had a median age of 37 years and a median CD4 count of 701 cells/mm^3^; 13.5% had detectable HIV-1 RNA. Floor effects were minimal, whereas ceiling effects were pronounced for role functioning and social functioning and notable for physical functioning and pain. Internal consistency was acceptable across all multi-item domains (Cronbach’s *α*, 0.770–0.924), and test–retest reliability was satisfactory [ICC (2,1), 0.708–0.850]. A parsimonious two-factor domain-level structure was supported. Clinically, comorbidity, CD4 < 500 cells/mm^3^, and a history of AIDS-defining illness were associated with poorer scores in selected domains, whereas scores did not differ by tablet burden.

**Conclusion:**

The Turkish MOS-HIV showed acceptable reliability and structural validity in a young, predominantly male, virologically suppressed population in urban tertiary care, and distinguished HRQoL differences by comorbidity, CD4 status, and history of AIDS-defining illness but not by tablet burden. Evaluation in women, older PLWH and more clinically heterogeneous populations is needed before broader use.

## 1 Introduction

By the end of 2024, an estimated 40.8 million people were living with HIV (PLWH) worldwide (1). In Türkiye, where the epidemic has expanded more recently, national surveillance reports have shown a continuing upward trend in both reported HIV diagnoses and the cumulative number of diagnosed cases. In 2025, the Ministry of Health reported a cumulative total of 51,469 HIV diagnoses, including 2,577 AIDS cases (2). In settings where treatment is universally accessible, combination antiretroviral therapy (ART) has transformed HIV from a fatal disease into a chronic manageable condition. With effective therapy, PLWH may achieve life expectancy approaching that of the general population (3). However, they continue to report substantially poorer health-related quality of life (HRQoL) than the general population, even in settings where most individuals have virological suppression and are immunologically stable (4). Available evidence suggests that, beyond the infection itself, HRQoL is shaped by social circumstances, relationship issues, comorbidities, and stigma (5). From this perspective, HRQoL may be understood as an individual’s perceived functioning across physical, emotional, psychological, and social domains of health (6).

Accordingly, achieving a high level of HRQoL has become an important goal and an essential component of HIV/AIDS care (7). In 2016, Lazarus and colleagues proposed complementing the Joint United Nations Program on HIV/AIDS (UNAIDS) 90–90–90 treatment cascade with a fourth “90” focused on HRQoL among virally suppressed individuals (8, 9). This fourth 90 target specifies that 90% of PLWH who achieve viral suppression should also have good HRQoL. In this framework, HRQoL is positioned as a core patient-centered outcome alongside viral suppression (10, 11). Subsequently, the testing–treatment–viral suppression cascade targets were increased to 95–95–95. In the 2021 UN General Assembly Political Declaration on HIV and AIDS, Member States committed to achieving the 95–95–95 targets by 2025: 95% of all PLWH know their status, 95% of those diagnosed receive antiretroviral therapy, and 95% of those on treatment attain viral suppression (12, 13). This renewed emphasis on outcomes beyond survival and virological control further underscores the need for rigorous measurement in both routine clinical care and intervention evaluation (14). Accordingly, HIV care requires instruments that not only perform well psychometrically but also identify clinically relevant residual burden in treated populations. Among HIV-specific patient-reported outcome measures (PROMs) assessing HRQoL, the Medical Outcomes Study HIV Health Survey (MOS-HIV) is one of the earliest and most widely used instruments, with substantial psychometric evaluation (15, 16). In a concise review of HRQoL PROMs for PLWH with fewer than 40 items, Cooper et al. identified the MOS-HIV as the most well-established measure (14). Similarly, Wen et al. (15) reported that, relative to other HIV-specific and generic PROMs, the MOS-HIV demonstrates relatively strong psychometric performance. The MOS-HIV is a brief, comprehensive HRQoL measure comprising 35 items across 10 health-status dimensions and one health transition item (16, 17).

Evidence on HRQoL among Turkish PLWH remains limited. To date, only the WHOQOL-HIV BREF and PoZQOL have been translated and validated for use in Turkish (18, 19). These instruments address quality of life broadly, including environmental and spiritual domains. The MOS-HIV, by contrast, is a health-status measure focused on physical and mental well-being, yields norm-based physical and mental health summary scores that allow comparison with the extensive international literature in which it has been used, and has the strongest psychometric evidence among HIV-specific PROMs (14, 15). Tükenmez Tigen et al. examined correlations and agreement between the Turkish MOS-HIV and the SF-36 using Bland–Altman analysis in a single-center sample of 114 PLWH (20); however, that study did not assess internal consistency, test–retest reliability, factorial structure, item-level scaling properties, measurement invariance, or known-groups validity. Consequently, the psychometric adequacy and clinical utility of an HIV-specific HRQoL instrument for Turkish-speaking PLWH have not been adequately demonstrated.

In contemporary HIV care, where virological suppression is increasingly achievable, detecting persistent impairments in physical, mental, and social well-being has become a clinical priority. We therefore conducted a multicenter psychometric evaluation of the Turkish MOS-HIV in a sample of ART-experienced PLWH, examining whether the instrument provides sufficiently reliable, valid, and interpretable scores for use in this population, including its ability to discriminate between clinically relevant subgroups.

## 2 Materials and methods

### 2.1 Study design and participants

Clinicians at five tertiary training and research hospitals in Istanbul recruited PLWH attending routine clinic visits for participation in this nonrandomized study between February and August 2025. Inclusion criteria were age ≥18 years, confirmed HIV infection, and ability to read Turkish. Exclusion criteria included inability to complete the questionnaires independently. Sociodemographic characteristics (age, sex, education, marital status and monthly household income) were obtained from participants by a brief self-report questionnaire completed at enrolment. Self-reported monthly household income was classified relative to Türkiye’s gross domestic product per capita as a benchmark for national average income. Participants reporting income above this benchmark were classified as high income and those at or below it as low/moderate income. Clinical data (CD4^+^ T-cell count and percentage, HIV-1 RNA, time since diagnosis, ART duration and regimen, comorbidities and history of AIDS-defining illness) were extracted from the clinical record. Detectable viral load was defined as HIV-1 RNA > 50 copies/mL. Mode of HIV acquisition and sexual orientation were not asked, because of their sensitivity in the local context. Comorbidity was defined as the presence of at least one physician-diagnosed condition. For structural validity analyses, sample size planning followed COSMIN study design guidance for factor analysis; accordingly, we targeted approximately seven participants per item (21).

### 2.2 Instrument

The Turkish version of the Medical Outcomes Study HIV Health Survey (MOS-HIV) was obtained through authorized distribution from Mapi Research Trust/ePROVIDE under User Agreement Special Terms No. 107150. Permission was granted for use of the instrument in this clinical research study. A review copy of the MOS-HIV and information on access are available through the official ePROVIDE/Mapi Research Trust instrument page (22). Participants evaluated clarity and comprehensibility using standard cognitive interviewing methods (23). Cognitive debriefing was conducted with 20 PLWH to assess the comprehensibility and interpretability of the Turkish MOS-HIV. These participants took part only in the debriefing phase and were not enrolled in the subsequent psychometric evaluation. They were adult PLWH able to read Turkish who were attending the participating clinics; their sociodemographic and clinical characteristics were not systematically recorded, because the purpose of this phase was to test the comprehensibility of the wording rather than to characterize the sample. The number of participants was set in advance in line with guidance that cognitive interviews are conducted in rounds of approximately 5–15 respondents, with additional rounds used only to re-test items revised after the previous round (24); a single round of 20 was therefore sufficient. Interviews followed a standardized cognitive interviewing approach that combined concurrent think-aloud techniques with verbal probing to identify potential ambiguity or culturally non-equivalent wording. Participants rated the comprehensibility of each item individually; items were considered acceptable if at least 80% of participants rated them as comprehensible according to the prespecified criterion. Interviewers recorded participant feedback throughout the process. Because all items met the prespecified comprehensibility threshold (>80%) and probing did not identify consistent misinterpretations or culturally incongruent meanings, no wording changes were made, and no further round was required.

The 35-item Medical Outcomes Study HIV Health Survey (MOS-HIV), a brief self-report instrument developed to assess HRQoL over the previous 4 weeks in PLWH, was used in this study. The MOS-HIV measures 10 health status domains: general health perceptions (GHP), physical functioning (PF), role functioning (RF), pain (PN), social functioning (SF), mental health (MH), energy/fatigue (EF), health distress (HD), cognitive functioning (CF), and quality of life (QOL), as well as a separate one-item health transition (HT) measure. Scoring followed the MOS-HIV User’s Manual without modification. Briefly, item responses were reviewed for out-of-range responses, and the 11 items designated in the manual were reverse scored so that higher values uniformly indicate better health. Raw domain scores were calculated as the sum of final item values within each domain and then linearly transformed to a 0–100 scale using the manual’s domain-specific transformation formulas, with higher transformed scores indicating better health status. No MOS-HIV item responses were missing; therefore, no within-domain imputation was required. Physical Health Summary (PHS) and Mental Health Summary (MHS) scores were calculated according to the manual’s algorithm using the 10 transformed domain scores. Specifically, each domain was standardized to the manual’s reference population, multiplied by the published PHS and MHS scoring coefficients, summed, and then transformed into *T*-scores (25). For reproducibility assessment, retests were offered to consecutive participants until the planned number of completed retests was exceeded. The number of participants required for a 95% confidence interval of total width 0.20 around an ICC of 0.70 with two administrations, estimated using Bonett’s formula, was 104 (26). A subsample of 105 participants completed MOS-HIV on two occasions separated by a median interval of 15 days. The interval was chosen to balance the two competing requirements for test–retest assessment: it had to be long enough that participants were unlikely to recall their initial responses, and short enough that the construct was unlikely to have changed (27).

### 2.3 Statistical analysis

Statistical analyses were performed using R (version 4.5.1; R Foundation for Statistical Computing, Vienna, Austria). Participant characteristics and MOS-HIV scale distributions were summarized using mean ± standard deviation (SD) or median (Q1–Q3), as appropriate, and categorical variables were summarized as counts and percentages. Summary scores were computed with the manual’s algorithm: domain scores were standardized against the manual’s reference sample, weighted by the published PHS and MHS scoring coefficients, and rescaled to a mean of 50 and SD of 10 (norm-based *T*-scores) (28). Floor and ceiling effects were considered substantial when more than 15% of participants scored at the minimum or maximum possible value (29). Internal consistency of multi-item MOS-HIV domains was evaluated using Cronbach’s *α*, with *α* ≥ 0.70 considered acceptable. Because SF, QOL, and HT are single-item scales, Cronbach’s *α* was not computed for these domains. Test–retest reliability was assessed using the intraclass correlation coefficient [ICC (1,2)], reported with 95% confidence intervals; ICC ≥ 0.70 was prespecified as evidence of adequate temporal stability (27). Comparability of participants who did and did not complete the retest was assessed with standardized mean differences, with standardised mean difference (|SMD|) < 0.20 taken as negligible (30).

Structural validity was tested by confirmatory factor analysis (CFA) of the 10 domain scores as indicators of two correlated factors, physical and mental health, estimated by robust maximum likelihood (MLR). Acceptable fit was defined as Comparative Fit Index (CFI) and Tucker–Lewis Index (TLI) ≥ 0.90, Root Mean Square Error of Approximation (RMSEA), and Standardized Root Mean Square Residual (SRMR) ≤ 0.08 (90% confidence interval reported for RMSEA) (31). Three two-factor models were prespecified and compared. Model A allowed GHP, EF and SF to load on both factors, following the manual’s PHS/MHS scoring (25, 28), Model B was a simple structure with no cross-loadings; and Model C allowed only the GHP cross-loading (32).

Item-level convergent and discriminant validity were evaluated using corrected item–scale correlations, with *r* ≥ 0.40 supporting convergence, and scaling success rates, with >90% considered strong (33). Measurement invariance across education groups (≤high school vs. university) was tested by multi-group CFA under the same specification, using ΔCFI ≤0.01 to judge metric and scalar invariance (34).

Known-group comparisons were assessed with Welch’s *t*-tests comparing domain and summary scores across four clinically meaningful strata: comorbidity (≥1 vs. none), CD4 category (<500 vs. ≥ 500 cells/mm^3^), history of AIDS-defining illness (yes vs. no), and tablet burden (single-tablet vs. multiple-tablet regimen). Lower scores, particularly in physical and symptom-related domains, were hypothesized *a priori* for comorbidity, lower CD4 counts, or a history of AIDS-defining illness; no direction was specified for tablet burden.

Ethics approval was obtained from the Haydarpaşa Numune Training and Research Hospital Ethical Review Board (HNEAH-BAEK 2024/165) and was conducted in accordance with the Declaration of Helsinki. All participants provided informed written consent prior to data collection.

## 3 Results

A total of 244 participants were included in the analysis. The median age was 37 years. The median time since HIV diagnosis was 54.4 months, and the median duration of ART was 55 months. The median CD4^+^ T-cell count was 701 cells/mm^3^, and the median CD4^+^ percentage was 34.5%. Detectable HIV-1 RNA was observed in 13.5% of participants. At least one comorbidity was present in 23.8% of participants, and 3.7% had a history of an AIDS-defining illness. The vast majority of participants were male. Educational attainment was relatively balanced between participants with university education and those with high school education or below. Most participants were single and reported low to moderate income (Table 1).

**Table 1:** Sociodemographic and clinical characteristics of participants.

Variable	Median (Q1–Q3) or *n* (%)
Age, years	37 (30–46)
Duration of HIV diagnosis, months	54.4 (28.9–85.6)
Duration of ART, months	55 (29.5–80.5)
CD4^+^ T-cell count, cells/mm^3^	701 (518.8–1016.8)
CD4^+^ percentage, %	34.5 (26–41)
Detectable HIV-1 RNA	33 (13.5%)
Presence of ≥1 comorbidity	58 (23.8%)
AIDS-defining illness	9 (3.7%)
Educational attainment	
University or higher	126 (51.6%)
≤High school	118 (48.4%)
Sex	
Male	222 (91.0%)
Female	22 (9.0%)
Marital status	
Married	69 (28.3%)
Single	175 (71.7%)
Income level	
High^a^	55 (22.5%)
Low/moderate	189 (77.5%)

ART, antiretroviral therapy; HIV-1 RNA, human immunodeficiency virus type 1 ribonucleic acid; CD4, cluster of differentiation 4.

a High = self-reported monthly household income above Türkiye’s GDP per capita (2025) (48).

Domain means and floor/ceiling percentages are shown in Table 2. Floor effects stayed below the 15% criterion in every domain, whereas ceiling effects exceeded it in the functioning and symptom domains (RF, SF, PF, PN, HD and CF) but not in the perception- and affect-based domains (MH, EF, QOL, GHP).

**Table 2:** Descriptive statistics and floor/ceiling effects for MOS-HIV domain and summary scores.

Domain scores (0–100)	Mean ± SD	Floor effect (%)	Ceiling effect (%)
General health perceptions	61.17 ± 25.63	0.4	8.2
Physical functioning	86.92 ± 18.41	0	48.8
Role functioning	89.75 ± 27.55	7	86.5
Pain	79.6 ± 20.5	0.4	33.6
Social functioning	86.56 ± 24.2	1.2	69.3
Mental health	61.15 ± 21.72	0.4	3.3
Energy/fatigue	58.61 ± 23.11	0.4	3.3
Health distress	73.22 ± 27.39	1.6	25
Cognitive functioning	74.55 ± 22.81	0.4	17.2
Quality of life	63.83 ± 21.17	0	13.9
Health transition	57.89 ± 23.61	3.3	13.1
Physical health summary	54.49 ± 8.47	—	—
Mental health summary	46.77 ± 11.78	—	—

Physical and mental health summary scores are norm-based *T*-scores computed with the user’s manual algorithm and are not on the 0–100 metric of the domain scores; floor and ceiling effects are therefore not applicable (—).

Cronbach’s *α* met the 0.70 criterion for all eight multi-item domains, ranging from 0.77 (PN) to 0.92 (HD) (Table 3). Inter-scale Pearson correlations were all positive (0.11–0.79). They were strongest among the mental-health-related domains (MH–EF 0.79, GHP–QOL 0.75, GHP–HD 0.72, EF–QOL 0.71) and moderate among the physical-health-related domains (PF–PN 0.63, PF–GHP 0.62, PF–RF 0.58); correlations between physical and mental health-related domains ranged from 0.32 to 0.60. HT correlated weakly with all other domains (0.11–0.46). No correlation exceeded 0.80 (Table 3).

**Table 3:** Inter-scale correlations and Cronbach’s alpha coefficients for the Turkish MOS-HIV domains.

Domain	Cronbach’s *α*	GHP	PF	RF	PN	SF	MH	EF	HD	CF	QOL	HT
General Health Perceptions	0.872	–										
Physical functioning	0.854	0.624	–									
Role functioning	0.789	0.472	0.580	–								
Pain	0.770	0.646	0.626	0.462	–							
Social functioning	—	0.472	0.551	0.496	0.457	–						
Mental health	0.871	0.714	0.487	0.410	0.533	0.406	–					
Energy/fatigue	0.848	0.711	0.540	0.427	0.569	0.398	0.793	–				
Health distress	0.924	0.718	0.467	0.457	0.516	0.482	0.666	0.697	–			
Cognitive functioning	0.883	0.552	0.398	0.361	0.421	0.323	0.658	0.647	0.586	–		
Quality of life	—	0.745	0.550	0.456	0.600	0.425	0.677	0.705	0.567	0.555	–	
Health transition	—	0.418	0.280	0.251	0.353	0.114	0.372	0.402	0.363	0.310	0.460	–

*n* = 244, — = not applicable (single-item domain). All correlations *p* < 0.01 except SF–HT (*p* = 0.08).

Retests were offered to consecutive participants until 100 were exceeded; 105 completed a second administration after a median of 15 days (IQR 13–16, range 11–20). Retest and non-retest participants did not differ materially in demographic, clinical or baseline MOS-HIV characteristics (all standardized mean differences ≤0.21; Supplementary Table 1). ICC (2,1) ranged from 0.71 (HT) to 0.85 (GHP) and the point estimates met the 0.70 criterion for all domains (Table 4).

**Table 4:** Test–retest reliability of MOS-HIV domains [ICC (2,1)].

Domain	ICC (2,1)	95% CI
General health perceptions	0.850	[0.786, 0.895]
Physical functioning	0.812	[0.735, 0.868]
Role functioning	0.833	[0.764, 0.883]
Pain	0.745	[0.646, 0.819]
Social functioning	0.718	[0.611, 0.799]
Mental health	0.797	[0.713, 0.858]
Energy/fatigue	0.747	[0.649, 0.821]
Health distress	0.771	[0.680, 0.838]
Cognitive functioning	0.713	[0.603, 0.796]
Quality of life	0.713	[0.605, 0.795]
Health transition	0.708	[0.599, 0.792]

ICC (2,1) = intraclass correlation coefficient from a two-way random-effects model for absolute agreement, single measurement.

Structural validity was evaluated by confirmatory factor analysis at the domain-score level comparing three prespecified two-factor models (Table 5). Model B fitted clearly worse than Models A and C. Because Model C fitted as well as Model A but with fewer parameters, the more parsimonious Model C was retained.

**Table 5:** Standardized factor loadings and fit of three two-factor confirmatory models of the Turkish MOS-HIV at the domain-score level.

Domain	Model A		Model B		Model C	
	Physical	Mental	Physical	Mental	Physical	Mental
Physical health-related domains						
Physical functioning	**0.816**	—	**0.726**	—	**0.823**	—
Role functioning	**0.666**	—	**0.589**	—	**0.666**	—
Pain	**0.767**	—	**0.743**	—	**0.768**	—
Social functioning	**0.714**	−0.072	—	**0.528**	**0.648**	—
Mental health-related domains						
Mental health	—	**0.865**	—	**0.861**	—	**0.868**
Energy/fatigue	−0.093	**0.964**	—	**0.881**	—	**0.885**
Health distress	—	**0.786**	—	**0.788**	—	**0.787**
Cognitive functioning	—	**0.715**	—	**0.715**	—	**0.718**
Quality of life	—	**0.803**	—	**0.804**	—	**0.801**
Shared domain						
General health perceptions	0.290	**0.614**	**0.890**	—	0.296	**0.613**
Model fit						
CFI	0.965		0.924		0.965	
TLI	0.949		0.899		0.953	
RMSEA (90% CI)	0.085 (0.062–0.109)		0.120 (0.099–0.142)		0.082 (0.059–0.105)	
SRMR	0.038		0.061		0.039	

Model A: structure underlying the PHS and MHS scoring algorithm, with general health perceptions, energy/fatigue and social functioning loading on both factors (28). Model B: simple structure with each domain on a single factor, general health perceptions assigned to physical and energy/fatigue and social functioning to the mental factor. Model C: structure reported for the Korean version, with social functioning on the physical factor, energy/fatigue on the mental factor and only general health perceptions loading on both (32). Loadings ≥ 0.30 in bold.

Corrected item-own-scale correlations for the multi-item domains ranged from 0.44 to 0.90, exceeding the prespecified criterion of *r* ≥ 0.40 for all items across domains. The strongest evidence of convergent validity was observed for HD (0.74–0.90), GHP (0.59–0.82), and CF (0.66–0.82). PF showed the lowest, although still adequate, range of item-own-scale correlations (0.44–0.81). Item-level discriminant validity was also supported. Scaling success rates reached 100% for GHP, PF, RF, PN, and CF, while MH (96.0%), EF (97.5%), and HD (97.5%) all exceeded the 90% threshold (Table 6). Scaling success metrics are not applicable to single-item domains.

**Table 6:** Item-level convergent and discriminant validity of the Turkish MOS-HIV domains.

Domain	Convergent validity	Discriminant validity	Convergent validity tests success rate	Discriminant validity tests success rate
General health perceptions	0.59–0.82	0.25–0.69	100	100
Physical functioning	0.44–0.81	0.14–0.56	100	100
Role functioning	0.66–0.66	0.21–0.55	100	100
Pain	0.7–0.7	0.33–0.66	100	100
Social functioning	—	0.11–0.55	—	100
Mental health	0.64–0.76	0.22–0.72	100	96
Energy/fatigue	0.58–0.75	0.23–0.76	100	97.5
Health distress	0.74–0.9	0.3–0.75	100	97.5
Cognitive functioning	0.66–0.82	0.2–0.64	100	100
Quality of life	—	0.42–0.74	—	100
Health transition	—	0.11–0.46	—	100

Multi-group CFA across education strata for the retained Model C supported metric and scalar invariance at the domain-score level according to the prespecified ΔCFI ≤ 0.01 criterion (ΔCFI for metric invariance = −0.002; ΔCFI for scalar invariance = −0.003). Although absolute fit indices were less favorable, incremental fit changed minimally across increasingly constrained models (configural CFI = 0.954, metric CFI = 0.953, and scalar CFI = 0.950). RMSEA remained in the borderline range (0.094–0.088), while SRMR increased modestly from 0.046 in the configural model to 0.060 in the metric model and 0.063 in the scalar model. Overall, these findings suggest that factor loadings and intercepts were sufficiently comparable across education groups to support cautious comparisons of latent physical and mental health factors derived from the 10 transformed domain scores.

Known-group comparisons revealed a consistent pattern in which clinical burden was most strongly reflected in physical health-related domains. Participants with at least one comorbidity scored significantly lower on GHP, PF, RF, PN, QOL, and PHS, whereas MH, EF, CF, SF, HT, and MHS did not differ significantly between groups; HD showed a borderline difference (Supplementary Table 2). Lower CD4 counts (<500 cells/mm^3^) were associated with significantly lower RF, SF, and PHS scores, with no significant differences in the remaining domains (Supplementary Table 3). A history of AIDS-defining illness was associated with lower SF and QOL scores; although the remaining comparisons did not reach statistical significance, effect sizes suggested potentially meaningful differences across several physical health-related domains, including GHP, PF, RF, PN, and PHS (Supplementary Table 4). No significant differences were observed between multiple-tablet and single-tablet regimens for any domain or summary score (Supplementary Table 5).

## 4 Discussion

The present study showed that the Turkish MOS-HIV had acceptable psychometric properties in an ART-experienced, clinically stable cohort. Internal consistency met the prespecified ≥0.70 criterion across all multi-item domains, test–retest ICCs met the criterion for all domains although with limited precision for seven of them, and item-scale convergent and discriminant validity supported appropriate item placement. The domain-level two-factor structure was broadly consistent with prior cross-cultural validations of the instrument (32, 35–40). Single-item scales (SF, QOL, and HT) were evaluated using test–retest reliability and construct validity, as internal consistency is not applicable to these domains (37).

Our sample was predominantly male, highly educated, and virologically well controlled—characteristics previously associated with higher HRQoL scores and greater reliability in selected domains (32, 36), but also with an increased risk of ceiling effects in functioning domains (37, 41). Approximately one-quarter of participants reported at least one comorbidity, which remained an important determinant of HRQoL burden (42, 43). Taken together, these sample characteristics support interpretation of the findings in a contemporary treatment setting, while indicating that they are most directly generalizable to urban, predominantly male, highly educated PLWH with largely suppressed viral load.

The score profile is consistent with the composition of the sample: young, ART-treated and largely virologically suppressed PLWH, in whom physical limitation is uncommon but fatigue, low mood and reduced perceived quality of life persist. Relative to the manual’s reference population, which was assembled before modern ART, our participants scored higher on PF, RF and PN and lower on MH, CF and QOL, so that the physical summary score lay above and the mental summary score below the reference mean of 50 (25). This divergence is the pattern anticipated by the “fourth 90” framework, in which viral suppression does not guarantee good HRQoL (8, 9), and it identifies mental health and energy rather than physical function as the domains most in need of clinical attention in this population.

Pronounced ceiling effects, particularly in RF and SF, warrant careful consideration. In the current treatment era, many PLWH receiving effective ART achieve good overall health and prolonged survival, yet physical, mental, and social burdens may persist despite virological control (3–5, 8). This distinction between viral suppression and optimal lived well-being, which is central to the “fourth 90” framework (8, 9), underscores the need for instruments that can discriminate adequately at higher levels of functioning. Ceiling effects may reduce this discriminative capacity and limit sensitivity to further improvement in well-controlled populations. Previous validation studies have reported similar patterns, particularly in PF and RF (36, 38), although the magnitude of these effects varies across studies and may reflect differences in clinical context, immunologic status, and ART regimens. In practice, RF, SF and PF will discriminate poorly among well-functioning patients and will be insensitive to further improvement, so responsiveness studies and individual monitoring in comparable populations should rely mainly on the mental-health-related domains and the summary scores.

Test–retest reliability was estimated in a subsample sized *a priori* to estimate an ICC of 0.70 with a 95% confidence interval of width 0.20. For domains whose ICC lies close to the criterion the interval therefore necessarily extends below 0.70; this was the case for seven domains (ICC 0.71–0.77), whereas GHP, PF, RF and MH (ICC 0.80–0.85) met the criterion across their entire interval. The weakest estimates were for the single-item domains (SF, QOL, HT) and PN; single-item scores carry more measurement error than multi-item scores, and pain and health transition are states that can genuinely change within 2 weeks, so lower stability for these domains is expected rather than a deficiency of the translation (27).

At the measurement level, the retained-CFA supported the correlated two-factor structure of physical and mental health that underlies the PHS and MHS summary scores, a structure first established by factor analysis in more than 2,500 patients with HIV disease (28) and since supported in several cross-cultural validations, though not previously tested in Turkish PLWH. Because the placement of GHP, EF and SF has varied across these validations, we compared three prespecified structures rather than imposing one (Table 5). Model C fitted as well as the manual-consistent Model A but with two fewer parameters, and clearly better than the simple-structure Model B; its only cross-loading, for GHP, is consistent with the broad content of general health perceptions, while PF, PN, RF and SF defined the physical factor and MH, EF, HD, CF and QOL the mental factor. RMSEA was borderline (0.082) but CFI, TLI and SRMR met conventional criteria, so the structure is a reasonable if not exact representation; residual misspecification may reflect minor uncaptured cross-loadings or distributional features such as ceiling effects in selected domains (44).

This pattern matches with previous MOS-HIV validations, in which the pacement of GHP, EF, and SF has been least consistent. In a Chinese validation using CFA, EF, general health, and SF contributed to both summary scores (37). Similarly, Henderson et al. (39) described EF and SF as “complex” domains that could load on both factors depending on clinical context, underscoring that mixed-loading behavior may not be uniform across samples and supporting the value of comparing prespecified specifications before selecting a final structure. Lau et al. (40) likewise identified two summary factors and reported that general health and EF loaded more strongly on MHS but also contributed to PHS, consistent with partially shared variance between the summary constructs. In our sample, EF and SF behaved predominantly as mental and physical indicators, respectively, whereas GHP retained a meaningful cross-loading.

Multi-group CFA across education strata supported metric and scalar invariance for the retained Model C, suggesting that factor loadings and intercepts were sufficiently comparable at the domain-score level to allow cautious comparisons of the latent physical and mental health factors across education groups. However, because absolute fit remained borderline (RMSEA 0.088–0.094 across steps), such comparisons should be interpreted cautiously.

Item-scale structure was supported by multitrait scaling analyses. Corrected item-own-scale correlations met the prespecified convergence criterion (*r* ≥ 0.40) for all items in the multi-item domains, and discriminant scaling success was high (96–100%). These findings indicate that items generally correlated more strongly with their intended domain than with competing domains, consistent with prior validation studies (36–38).

Evidence regarding associations between CD4 count and HRQoL remains heterogeneous in the literature. The limited number of domains that differentiated by CD4 category in our cohort may reflect the high level of ART exposure and generally well-controlled clinical status of the sample, together with ceiling effects in functioning domains that may reduce discrimination at the upper end of the score distribution (35, 40). The concentration of comorbidity-related differences in physical and symptom-related domains, rather than mental health domains, is consistent with previous evidence linking comorbidity burden primarily to physical HRQoL impairment in PLWH (41, 45) and provides supportive evidence of known-groups validity for the Turkish MOS-HIV (42, 43). Although only SF and QOL reached statistical significance in the AIDS-defining illness comparison, effect sizes across several physical health-related domains pointed in the expected direction, consistent with earlier work linking prior advanced HIV disease to poorer functioning and greater symptom burden (42). The small subgroup size (*n* = 9) likely limited statistical power for these comparisons. The absence of HRQoL differences by tablet burden is consistent with evidence that single-tablet regimens may improve treatment satisfaction without consistently translating into better HRQoL (46). Earlier studies reporting improved HRQoL after switching to single-tablet regimens (47) may partly reflect the act of simplification itself, rather than a sustained advantage of one regimen type over another. In our cross-sectional comparison, where participants were already established on their current regimens, such transient effects would not be captured, consistent with observations that regimen-type differences attenuate once patients are stably treated (46).

The composition of the sample bears directly on how the known-groups results should be read. Participants were young, predominantly male, ART-experienced and largely virologically suppressed, so the clinical contrasts available were few and the groups small. The differences observed, associated with comorbidity, lower CD4^+^ count and AIDS history, and concentrated in the physical health-related domains, show that the instrument registers physical morbidity in this population, but they do not establish that it discriminates between socially distinct groups, between women and men, or across the age range of PLWH, none of which could be tested. The psychometric properties reported here should therefore be regarded as established for the population studied and as preliminary for others. This study has several limitations. The design was cross-sectional, so the factor structure was established at a single time point and responsiveness, and minimal important differences remain unestablished. Participants were recruited from tertiary centers in a single city and place of residence was not recorded, so urban/rural differences could not be examined. The sample was predominantly male, young, ART-experienced and clinically well controlled, which precluded comparisons by sex or age, restricted the known-groups analyses to few contrasts, and limits generalizability to women, older PLWH, treatment-naïve individuals and those with advanced disease; mode of HIV acquisition and sexual orientation were not recorded. The ceiling effects discussed above may limit discrimination and responsiveness in the physical health-related domains, and the borderline RMSEA indicates some residual misfit of the retained model.

In conclusion, the Turkish MOS-HIV showed acceptable measurement performance in this young, predominantly male, urban, clinically stable sample of PLWH and identified clinically meaningful HRQoL differences related to comorbidity, lower CD4 count, and history of AIDS-defining illness. Its main value in similar populations may lie in structured assessment of residual patient-reported burden. Longitudinal responsiveness, thresholds for clinically important change, and performance in women, older PLWH and more clinically diverse populations remain to be established.

## Data Availability

The raw data supporting the conclusions of this article will be made available by the authors, without undue reservation.
