# Supplementary material for: Reliability and validity of the Turkish translation of the MOS-HIV health survey in people living with HIV
Source: Front Public Health. 2026 Oct 6;14:1966184. doi: 10.3389/fpubh.2026.1966184 (PMC13680415; doi:10.3389/fpubh.2026.1966184)
Supplement: Supplementary file 1 [file Supplementary_file_1.DOCX]

**Supplementary Table 1.** Characteristics of participants who did (n = 105) and did not (n = 139) complete the test–retest administration of the Turkish MOS-HIV.

| **Characteristic** | **No retest (n = 139)** | **Retest (n = 105)** | **SMD** |
| --- | --- | --- | --- |
| *Sociodemographic characteristics* | | | |
| Age, years | 39.6 ± 11.0 | 38.3 ± 10.1 | -0.12 |
| Male sex | 128 (92.1) | 94 (89.5) | -0.09 |
| University education or higher | 67 (48.2) | 59 (56.2) | +0.16 |
| Single | 99 (71.2) | 76 (72.4) | +0.03 |
| High income | 27 (19.4) | 28 (26.7) | +0.17 |
| *Clinical characteristics* | | | |
| Time since HIV diagnosis, months | 58.7 ± 45.4 | 60.3 ± 35.3 | +0.04 |
| Duration of ART, months | 57.6 ± 44.9 | 59.9 ± 31.9 | +0.06 |
| CD4⁺ T-cell count, cells/mm³ | 742 ± 348 | 816 ± 357 | +0.21 |
| CD4⁺ count < 500 cells/mm³ | 33 (23.7) | 22 (21.0) | -0.07 |
| Detectable HIV-1 RNA | 18 (12.9) | 15 (14.3) | +0.04 |
| ≥ 1 comorbidity | 36 (25.9) | 22 (21.0) | -0.12 |
| History of AIDS-defining illness | 7 (5.0) | 2 (1.9) | -0.17 |
| *Baseline MOS-HIV scores* | | | |
| General health perceptions | 60.6 ± 26.9 | 61.6 ± 24.0 | +0.04 |
| Physical functioning | 87.2 ± 18.7 | 86.2 ± 18.6 | -0.05 |
| Role functioning | 89.9 ± 26.4 | 89.0 ± 29.4 | -0.03 |
| Pain | 80.7 ± 20.9 | 77.5 ± 19.6 | -0.16 |
| Social functioning | 86.8 ± 23.8 | 86.1 ± 24.0 | -0.03 |
| Mental health | 61.7 ± 21.8 | 60.4 ± 22.2 | -0.06 |
| Energy/fatigue | 59.7 ± 22.8 | 57.7 ± 23.7 | -0.09 |
| Health distress | 73.5 ± 27.2 | 72.1 ± 27.9 | -0.05 |
| Cognitive functioning | 76.3 ± 20.7 | 72.1 ± 25.6 | -0.18 |
| Quality of life | 63.8 ± 22.4 | 63.1 ± 19.9 | -0.04 |
| Health transition | 60.1 ± 22.3 | 56.0 ± 25.8 | -0.17 |
| Physical health summary | 54.7 ± 8.6 | 54.1 ± 8.3 | -0.07 |
| Mental health summary | 47.1 ± 11.8 | 46.2 ± 12.0 | -0.07 |

**Note:** Values are mean ± SD or n (%). SMD, standardized mean difference (retest minus no-retest; Cohen's d for continuous variables, Cohen's h-equivalent for proportions), with |SMD| < 0.20 conventionally regarded as negligible. Retests were offered to consecutive participants in order of enrolment until 100 completed retests were exceeded; the retest was administered after a median of 15 days (IQR 13–16; range 11–20). High income = self-reported monthly household income above Türkiye's GDP per capita. PHS and MHS are norm-based T-scores (mean 50, SD 10); all other MOS-HIV scores are on the 0–100 metric. ART, antiretroviral therapy.

**Supplementary Table 2. Known-groups validity: MOS-HIV domain and item scores by comorbidity status.**

| MOS Scales | No Comorbidity (n=186) | Comorbidity (n=58) | p value | Cohen’s d |
| --- | --- | --- | --- | --- |
| General Health Perceptions | 63.5 ± 24.8 | 53.8 ± 27.2 | **0.018** | 0.38 |
| Physical Functioning | 88.7 ± 17.1 | 81.3 ± 21.4 | **0.019** | 0.4 |
| Role Functioning | 93.0 ± 22.2 | 79.3 ± 38.7 | **0.012** | 0.51 |
| Pain | 81.8 ± 19.2 | 72.4 ± 23.0 | **0.006** | 0.47 |
| Social Functioning | 88.1 ± 22.3 | 81.7 ± 29.1 | 0.131 | 0.26 |
| Mental Health | 62.3 ± 21.6 | 57.5 ± 21.9 | 0.15 | 0.22 |
| Energy/Fatigue | 59.8 ± 22.6 | 54.9 ± 24.4 | 0.183 | 0.21 |
| Health Distress | 75.3 ± 26.3 | 66.4 ± 29.9 | **0.043** | 0.33 |
| Cognitive Functioning | 74.8 ± 22.7 | 73.9 ± 23.3 | 0.801 | 0.04 |
| Quality of Life | 66.1 ± 21.0 | 56.5 ± 20.2 | **0.002** | 0.46 |
| Health Transition | 58.5 ± 23.4 | 56.0 ± 24.5 | 0.506 | 0.1 |
| Physical Health Summary | 55.5 ± 7.5 | 51.4 ± 10.4 | **0.007** | 0.49 |
| Mental Health Summary | 47.6 ± 11.6 | 44.1 ± 12.1 | 0.057 | 0.3 |

***Note:*** *Values are mean ± SD; domain scores are on the 0–100 metric and the physical and mental health summary scores (PHS, MHS) are norm-based T-scores; higher scores indicate better health-related quality of life. Values in bold denote p < 0.05.*

**Supplementary Table 3. Known-groups validity: MOS-HIV domain and item scores by CD4 category.**

| MOS Scales | CD4<500 (n=55) | CD4>=500 (n=189) | p value | Cohen’s d |
| --- | --- | --- | --- | --- |
| General Health Perceptions | 56.0 ± 28.8 | 62.7 ± 24.5 | 0.123 | -0.26 |
| Physical Functioning | 82.3 ± 24.7 | 88.3 ± 16.0 | 0.093 | -0.33 |
| Role Functioning | 81.8 ± 35.2 | 92.1 ± 24.5 | **0.047** | -0.38 |
| Pain | 75.2 ± 22.3 | 80.9 ± 19.8 | 0.089 | -0.28 |
| Social Functioning | 79.3 ± 30.8 | 88.7 ± 21.6 | **0.038** | -0.39 |
| Mental Health | 60.4 ± 21.7 | 61.4 ± 21.8 | 0.783 | -0.04 |
| Energy/Fatigue | 57.5 ± 24.4 | 58.9 ± 22.8 | 0.711 | -0.06 |
| Health Distress | 70.9 ± 28.4 | 73.9 ± 27.1 | 0.492 | -0.11 |
| Cognitive Functioning | 74.5 ± 22.9 | 74.6 ± 22.8 | 0.999 | 0 |
| Quality of Life | 60.9 ± 21.4 | 64.7 ± 21.1 | 0.251 | -0.18 |
| Health Transition | 58.2 ± 24.1 | 57.8 ± 23.5 | 0.918 | 0.02 |
| Physical Health Summary | 51.7 ± 11.5 | 55.3 ± 7.2 | **0.032** | -0.43 |
| Mental Health Summary | 45.8 ± 12.3 | 47.0 ± 11.6 | 0.515 | -0.1 |

***Note:*** *Values are mean ± SD; domain scores are on the 0–100 metric and the physical and mental health summary scores (PHS, MHS) are norm-based T-scores; higher scores indicate better health-related quality of life. Values in bold denote p < 0.05.*

**Supplementary Table 4. Known-groups validity: MOS-HIV domain and item scores by history of AIDS defining illness.**

| ****Characteristic**** | ****No History of AIDS Defining Illness (n**** = 235) | ****History of AIDS Defining Illness (n**** = 9) | ****p-value**** | Cohen’s d |
| --- | --- | --- | --- | --- |
| General Health Perceptions | 62 ± 25 | 43 ± 33 | 0.132 | 0.73 |
| Physical Functioning | 88 ± 17 | 68 ± 32 | 0.094 | 1.11 |
| Role Functioning | 91 ± 26 | 61 ± 49 | 0.104 | 1.10 |
| Pain | 80 ± 20 | 67 ± 32 | 0.245 | 0.66 |
| Social Functioning | 88 ± 22 | 53 ± 41 | **0.037** | 1.48 |
| Mental Health | 61 ± 22 | 60 ± 17 | 0.783 | 0.08 |
| Energy/Fatigue | 59 ± 23 | 57 ± 21 | 0.788 | 0.09 |
| Health Distress | 73 ± 28 | 69 ± 24 | 0.595 | 0.16 |
| Cognitive Functioning | 74 ± 23 | 79 ± 16 | 0.435 | -0.20 |
| Quality of Life | 64 ± 21 | 50 ± 18 | **0.042** | 0.68 |
| Health Transition | 58 ± 24 | 67 ± 18 | 0.168 | -0.39 |
| Physical Health Summary | 55 ± 8 | 44 ± 16 | 0.069 | 1.36 |
| Mental Health Summary | 47 ± 12 | 44 ± 10 | 0.418 | 0.24 |

***Note:*** *Values are mean ± SD; domain scores are on the 0–100 metric and the physical and mental health summary scores (PHS, MHS) are norm-based T-scores; higher scores indicate better health-related quality of life. Values in bold denote p < 0.05.*

**Supplementary Table 5. Known-groups validity: MOS-HIV domain and item scores by ART regimen.**

| ****Characteristic**** | Multiple Tablet Regimens ****(n**** = 62) | Single Tablet Regimens ****(n**** = 170) | ****p-value**** | Cohen’s d |
| --- | --- | --- | --- | --- |
| General Health Perceptions | 61 ± 26 | 60 ± 25 | 0.776 | 0.04 |
| Physical Functioning | 87 ± 18 | 87 ± 19 | 0.873 | 0.02 |
| Role Functioning | 91 ± 25 | 89 ± 29 | 0.601 | 0.07 |
| Pain | 80 ± 20 | 79 ± 21 | 0.555 | 0.09 |
| Social Functioning | 89 ± 21 | 86 ± 25 | 0.337 | 0.13 |
| Mental Health | 58 ± 25 | 62 ± 21 | 0.318 | -0.16 |
| Energy/Fatigue | 58 ± 23 | 59 ± 23 | 0.771 | -0.04 |
| Health Distress | 70 ± 30 | 74 ± 26 | 0.412 | -0.13 |
| Cognitive Functioning | 73 ± 25 | 75 ± 22 | 0.619 | -0.08 |
| Quality of Life | 66 ± 23 | 63 ± 21 | 0.352 | 0.15 |
| Health Transition | 58 ± 23 | 58 ± 23 | 0.937 | 0.01 |
| Physical Health Summary | 55 ± 8 | 54 ± 9 | 0.358 | 0.13 |
| Mental Health Summary | 46 ± 13 | 47 ± 11 | 0.649 | -0.07 |

***Note:*** *Values are mean ± SD; domain scores are on the 0–100 metric and the physical and mental health summary scores (PHS, MHS) are norm-based T-scores; higher scores indicate better health-related quality of life. Values in bold denote p < 0.05.*
